# Medical Staffing Models of Inpatient Major Trauma Services in Australia and New Zealand

**DOI:** 10.1111/1742-6723.70289

**Published:** 2026-06-19

**Authors:** Haania Abbasi, Elyssia M. Bourke, Mohsin Ejaz, Andrew Buck

**Affiliations:** ^1^ Department of Emergency Medicine Royal Melbourne Hospital Parkville Australia; ^2^ Department of Critical Care University of Melbourne Melbourne Australia; ^3^ Emergency Department Grampians Health Ballarat Australia; ^4^ Department of Intensive Care St Vincent's Hospital Melbourne Australia; ^5^ Monash Children's Hospital Clayton Australia

**Keywords:** emergency medicine, major trauma services, medical staffing, multidisciplinary care, trauma systems

## Abstract

**Objective:**

To quantify and describe the different medical specialty staffing models utilised by inpatient trauma services in Major Trauma Centres in Australia and New Zealand.

**Methods:**

A standardised telephone survey of all Major Trauma Centres (MTCs) in Australia and New Zealand was conducted between May and August 2024.

**Results:**

16/27 (67%) of MTC's in Australia utilise multi‐specialty staffing models for their inpatient trauma service. Two of the seven (29%) MTC's in New Zealand utilise a multi‐specialty staffing model.

**Conclusions:**

This study presents the results of a survey of all adult and paediatric major trauma centres in Australia and New Zealand. It quantifies and describes medical specialist staffing models of inpatient major trauma services. Two‐thirds of MTC's in Australia and almost one‐third of MTC's in New Zealand utilise multi‐specialty medical specialist staffing models for their inpatient trauma services. The range of non‐surgical specialties employed in these roles includes Emergency Physicians (FACEM), Anaesthetists (FANZCA) and Intensivists (FCICM). Of those services that utilise a multi‐specialty model, there is wide variation in the number and mix of specialties involved, as well as in the structure and function of the admitting services.

## 1 Introduction

In Australia and New Zealand, major trauma centres (MTC's) provide the full spectrum of definitive care to severely injured patients.

The Royal Australasian College of Surgeons (RACS) has partnered with the Australian and New Zealand College of Anaesthetists (ANZCA), the College of Intensive Care Medicine (CICM), the Australasian College for Emergency Medicine (ACEM) and the Australasian Trauma Society to publish the Model Resource Criteria For Trauma Services as part of the RACS Trauma Care Verification Program (TCVP), which benchmarks local trauma services against best practice care. Trauma facilities are categorised from Level I (which provide the highest level of trauma care) to Level IV (those able to provide basic trauma care only). These guidelines, last updated in April 2022, outline the requirements for accreditation for each level of trauma facility and the minimum standards required to be classified within each category (Appendix B). A rigorous, multi‐disciplinary, peer‐review accreditation process allows the standards to be upheld [1].

The Model Resource Criteria (MRC) does not define the term “Major Trauma Centre” but implies that Level I verification confers “Major Trauma Facility” status, whilst acknowledging that Level II hospitals should have identical clinical aspects of care to Level I centres and differ only in the non‐clinical verification criteria.

Each state/territory/region health system defines (and allocates patient flow to) Major Trauma Centres based on locally agreed state/territory/regional trauma system criteria. With few exceptions across Australia and New Zealand, all listed ‘Major Trauma Centres’ have obtained RACS Level I accreditation; however, in some regions there are Level II centres designated as MTC's, and RACS verification status can change over time. There is no set time for re‐verification, but review every 5 years has been suggested.

This study surveyed hospitals that are defined by their state/territory/regional trauma systems as ‘Major Trauma Centres’.

This tiered model of care enables all health services to manage trauma patients within the resources available at each centre, with a focus on patient‐centred outcomes [2]. A hub‐and‐spoke style system is utilised, which includes a pre‐hospital triage service that can re‐direct patients to the appropriate health service based on geographical travel times and injury severity [3]. Despite this overarching system, there is no clearly defined ideal nor gold standard model of medical staffing for MTC's.

MTC's in Australia and New Zealand have undergone an evolution with regard to medical specialist staffing over the last 15 years. Previously, inpatient trauma services were only staffed by surgeons, but many MTC's now utilise a multi‐specialty medical staffing model. To date, there is no published data detailing the specific staffing models employed by MTC's in Australasia.

Within the RACS Model Resource Criteria (MRC) accreditation guidelines, there are no recommendations regarding the optimal medical staffing model for MTC's, other than the requirement for a minimum of two consultant trauma surgeons to be working at a Level 1 centre. There is also no reference to medical specialties other than surgeons working in the trauma consultant role. Outside of verification information held privately by RACS or anecdotal descriptions of staffing models shared privately between interstate/trans‐Tasman colleagues or associates, there is no publicly available, easily accessible nor collated database of information regarding the current medical specialist staffing mix of MTC's in Australia and New Zealand.

Across other developed nations including the United Kingdom, the United States of America, and Canada, most MTC inpatient services are overseen by medical staff with a surgical fellowship. This has also been the traditional approach in Australia and New Zealand. However, in 2009 The Alfred Hospital in Melbourne introduced a multi‐specialty approach to the medical staffing of the inpatient trauma service and became the first MTC in Australasia to appoint an Emergency Physician (FACEM) as an inpatient trauma consultant and director of trauma [4]. Since then, several Australian and New Zealand MTC's have adopted a multi‐specialty staffing model for their inpatient trauma services. In addition to surgeons, they have appointed emergency physicians, anaesthetists, and intensivists to work as medical specialists on their inpatient trauma services. These positions have been referred to as ‘Trauma Physicians’, ‘Trauma Specialists’ or ‘Trauma Consultants’, however, these terms have not been defined, and there is currently no agreed single title that describes a non‐surgical medical specialist working for an inpatient trauma service at an MTC.

This project aimed to clarify the current models of care in Australia and New Zealand with a particular focus on the medical specialist staffing structure of each major trauma centre.

## 2 Method

A cross‐sectional observational survey of each MTC in Australia and New Zealand was performed, including adult and paediatric major trauma centres. Data was collected via a two‐step process; a questionnaire followed by reviewing the publicly available annual statistical report from the Australia and New Zealand Trauma Registry (ANZTR) for each individual major trauma centre [5]. The ANZTR data was included to provide perspective on the relative volume of trauma seen at each centre, and if there was any association between volume and staffing models. Unfortunately, no equivalent registry exists for New Zealand and hence this information has not been presented.

A telephone questionnaire was conducted with a member of the trauma team within each hospital, most commonly the hospital trauma coordinator. The questionnaire included four questions (Appendix A). The data were collected over a four‐month period between May and August 2024. We reviewed data from the ANZTR pertaining to the period between July 2021 and June 2022. The template data sheet can be found in Appendix B.

The following definitions are applied in the results section:

- *Multi‐specialty model:* refers to services where non‐surgeons are providing ward cover to admitted trauma patients, including via a ‘consulting’ trauma service model. Services with a surgical bed card and a non‐surgeon director who functions in a predominantly non‐clinical or non‐patient‐facing role have been recorded as ‘YES’ as this directorial position represent a significant role in the function of the major trauma service.
- *Inpatient trauma service specialty staffing/bed‐card:* refers to the type of bed card as well as medical specialties involved with direct patient care.
- Surgeons only = surgical bed card, only surgeons providing ward cover/clinical care.
- Surgeons + other specialties (e.g. FACEM, FANZCA, FCICM) = may have a surgical bed card, a trauma bed card, or other model (e.g., consulting service).

## 3 Results

To account for geographical and structural differences between the health systems across Australia and New Zealand, the findings have been summarised separately.

### 3.1 Australia

There are 27 Major Trauma Centres in Australia. Table 1 summarises the composition of each hospital's trauma service according to model of care and medical specialty staffing. There was no apparent relationship between relative volume of trauma received by a MTC and the medical staffing model.

**TABLE 1:** Medical specialist staffing of inpatient trauma services at Major Trauma Centres within Australia.

		% State trauma (ATR)	Case mix (adults/paediatrics/both)	Multi‐specialty model^a^	Inpatient trauma service specialty staffing/bed‐card	Director/co‐director specialty
*Australian Capital Territory*						
1	Canberra	100%	Both	YES	Surgeons, FACEM, FCICM	FCICM director
*New South Wales*						
2	John Hunter	19%	Adults	NO	Surgeons only	Surgeon director and co‐director
3	Liverpool	20%	Adults	NO	Surgeons only	Surgeon director and co‐director
4	Royal Northshore	11%	Adults	YES	FANZCA, FACEM	FANZCA director
5	Royal Prince Alfred	10%	Adults	YES	Surgeons, FACEM	FACEM director
6	St. Vincents	5%	Adults	YES	Surgeons only	Surgeon director FACEM co‐director
7	St. George	11%	Adults	NO	Surgeons only	Surgeon director
8	Westmead	17%	Adults	YES	Surgeons, FACEM, FANZCA	Surgeon director
9	John Hunter Children's	2%	Paediatric	NO	Surgeons only	Surgeon director
10	Sydney Children's	3%	Paediatric	YES	Surgeons only	FACEM director
11	Westmead Children's	3%	Paediatric	YES	FACEM only	FACEM director
*Northern Territory*						
12	Royal Darwin	100%	Both	YES	Surgeons, FACEM	FACEM director and co‐director
*Queensland*						
13	Gold Coast University	21%	Adults	YES	Surgeons, FACEM, FANZCA	Surgeon director
14	Princess Alexandra	NA Since 2018	Adults	YES	Surgeons, FACEM	Surgeon director
15	Sunshine Coast	21%	Adults	YES	Surgeons, FACEM	FACEM director
16	Townsville University	21%	Adults	NO	Surgeons only	Surgeon director
17	Royal Brisbane and Women's	30%	Adults	YES	Surgeons, FACEM	FACEM director and FACEM co‐director
18	Queensland Children's	7%	Paediatric	YES	Surgeons only	Surgeon director and FACEM co‐director
*South Australia*						
19	Flinders	20%	Adults	YES	Surgeons only	FACEM director
20	Royal Adelaide	72%	Adults	YES	Surgeons only	FACEM director^a^
21	Women and Children's	8%	Paediatrics	NO	Surgeons only	Surgeon director and co‐director
*Tasmania*						
22	Royal Hobart	100%	Both	YES	FACEM, FCICM, FANZCA	FANZCA director and FACEM co‐director
*Western Australia*						
23	Royal Perth	93%	Adults	NO	Surgeons only	Surgeon director and co‐director
24	Perth Children's	7%	Paediatric	YES	Surgeons only	FACEM director
*Victoria*						
25	Alfred	51%	Adults	YES	Surgeons, FACEM, FANZCA	FACEM director
26	Royal Melbourne	44%	Adults	NO	Surgeons only	Surgeon director
27	Royal Children's	5%	Paediatric	NO	Surgeons only	Surgeon director

Abbreviations: ATR, annual trauma registry; MST, multi‐specialty team; FACEM, emergency physician; FANZCA, anaesthetist; FCICM, intensivist.

^a^ Information taken from Australian Trauma Registry reports.

Overall, 18 of the 27 (67%) trauma services are using a multi‐specialty model, defined as having non‐surgeons in a patient‐facing (ward cover) and/or director or deputy/co‐director role/s. The remaining nine (33%) are not using a multi‐specialty model.

Fifteen of the 27 (55%) trauma services have a surgical bed card and/or solely surgeons providing ward cover. The remaining 12 MTC's (44%) have multiple specialties providing ward cover or admission under a non‐surgical/dedicated trauma bed card. Fifteen of the 27 services (55%) have a non‐surgeon in a director or deputy/co‐director role. The majority of these (13/15, 87%) are FACEMs. There was one service with a FANZCA director and one with a FCICM director. Six of the 15 trauma services (40%) that had a surgical‐only model have a non‐surgeon in a director or deputy/co‐director role, all of whom are FACEMs.

### 3.2 New Zealand

There are seven Major Trauma Centres in New Zealand. Table 2 summarises the composition of each hospital's trauma service according to model of care and medical specialty staffing.

**TABLE 2:** Medical specialist staffing of inpatient trauma services at Major Trauma Centres within New Zealand.

		Case mix (adults/paediatrics/both)	Multi‐specialty model	Inpatient trauma service specialty staffing/bed‐card	Director/co‐director specialty
*North Island: Northern Region*					
1	Auckland City	Adults	YES	Surgeons, FACEM, FANZCA	Surgeon director
2	Middlemore	Adults	NO	Surgeons only	Surgeon director
3	Starship	Paediatric	NO	Surgeons only	Surgeon director
*North Island: Midland Region*					
4	Waikato	Adults	NO	Surgeons only	Surgeon director and co‐director
*North Island: Central Region*					
5	Wellington Regional	Adults	NO	Surgeons only	Surgeon director and co‐director
*South Island*					
6	Christchurch	Both	NO	Surgeons only	Surgeon director
7	Dunedin	Adults	YES	Surgeons only	Surgeon director FACEM co‐director

In New Zealand, six of the seven (86%) MTCs have a surgical bed card and/or surgeons only providing ward cover. Two (29%) are using a multi‐specialty model. Five (71%) have a surgical bed card and/or surgeons only providing ward cover and surgeons only in director or deputy/co‐director roles. Auckland City Hospital and Waikato Hospital utilise trauma bed cards, while all other major trauma centres admit patients under a surgical bed card. Consultant ward staffing varies, with Auckland City Hospital adopting a multi‐specialty team (MST) approach to ward rounds while the remaining six major trauma centres rely solely on surgeons for this role. Leadership is predominantly surgical, and six out of the seven major trauma centres have surgeons serving as directors, co‐directors, or both.

## 4 Discussion

These findings highlight the significant variability in the structure and function of MTCs across Australia and New Zealand. Although some hospitals are implementing a multi‐specialty approach to managing trauma inpatients, the leadership roles and staffing models differ considerably between centres.

These differences highlight the need to evaluate how these various staffing models may impact service delivery and patient outcomes. There are currently no published gold‐standard staffing models, guidelines nor recommendations for MTC staffing. The reasons for this are unclear but may be due to a lack of robust evidence to guide change, a lack of expert consensus, or both. International evidence suggests the integration of multi‐specialty teams improves patient outcomes, including reduced mortality and ICU admissions [6]. Hence, a concerted focus on the creation of Australasian evidence to determine if this model should be recommended is required.

The wide variability in service design and delivery has made allocating defined labels or categories challenging. Some patterns were noted with regard to service design/delivery and specialties which provide direct clinical care, with four main models identified:

1. Surgical bed card, surgeons delivering clinical care
2. Surgical bed card, multi‐specialty consulting trauma service delivering clinical care
3. Trauma bed card, surgeons delivering clinical care
4. Trauma bed card, multi‐specialty clinical care.

Different director/co‐director models were identified, with a mix of surgeons and other medical specialists in director roles (all of whom provided some form of direct clinical care), and surgeons and other specialties in co‐director (or deputy director) roles, with some providing direct clinical care, whilst some held a purely administrative role within the trauma service.

In countries with a high incidence of blunt trauma, such as Australia and New Zealand, a significant portion of non‐orthopaedic and non‐neurosurgical trauma is now managed non‐operatively [7]. While surgical intervention remains essential for many trauma patients, and the involvement of surgeons in leading or supporting trauma services is invaluable, integrating non‐surgical medical staff into inpatient trauma teams may offer additional benefits. A multidisciplinary approach may enhance initial trauma resuscitation, while beyond the resuscitation phase, non‐surgical specialists can contribute to perioperative assessment, pain management, and the treatment of intoxication and withdrawal [8]. However, there is currently no local data or published research evaluating the advantages of non‐surgeons in a multi‐specialty trauma care model in Australia and New Zealand. Further research is needed to better understand the specific skills and contributions these specialties bring to inpatient trauma services.

The variability of staffing models identified in this study coupled with limited formalised modes of communication and knowledge sharing systems between interstate and trans‐Tasman major trauma centres highlights the absence of standardised approaches to staffing and service delivery. The reasons for this variability are not known but may stem from state or region‐based health administration and funding, the decentralised governance of major trauma centres, and the unique geographical and healthcare needs of each region.

## 5 Conclusion

This study is the first to describe the structure of medical staffing models for inpatient trauma services of MTCs in Australia and New Zealand and reveals diversity in the approach to trauma service delivery. Whilst a multi‐specialty team approach shows promise based on international data, and a majority of services in Australia utilise a multi‐specialty model, the local impact of having non‐surgeons staffing trauma services remains underexplored.

To the best of our knowledge, all Levels 1 and 2 trauma centres across Australasia have been included in this work. The hospitals surveyed were determined by the authorship team, who have extensive connections with the trauma networks in all states and territories of Australia and New Zealand. However, RACS does not publish a list of the Levels of each trauma centre. Hence, to prevent inaccurate characterisation of each centre, we have not provided the individual Level of each hospital in this work.

Future research should focus on evaluating the specific knowledge and skill‐sets that different specialties bring to inpatient trauma services and the actual or potential clinical benefits to patients that health services may derive from a multi‐specialty model. Given the wide range of variables that impact on clinical outcomes, linking these benefits to staffing models may prove difficult. By documenting and publishing trauma service staffing models, it is hoped that trauma services may find it easier to engage in collaboration and communication between major trauma centres, and that it will facilitate the sharing of information with regard to optimal trauma service design and delivery, as well as acting as a foundation for future research in this field.

## Conflicts of Interest

The authors declare no conflicts of interest.

## Data Availability

All data generated or analysed during this study are included in this published article.

## Appendix A See Table A1

**TABLE A1:** Telephone questionnaire used in this study.

*Telephone questionnaire*: Trauma services in Australia and New Zealand. 1. Is there an admitting trauma service/which bed‐card are trauma patients admitted under? e.g. Trauma, Surgery, Other. 2. What is the sub‐specialty of the consultants working as the primary inpatient Trauma Consultants? e.g. Combination, Surgeon, Emergency Physician, Anaesthetists, Intensivist, other. 3. What are the sub‐specialty qualification(s) of the Director and Co/Deputy‐Director of Trauma? This question allows us to understand if there is a difference in outcomes based on the subspecialty of the lead. 4. Are there any research/special programmes that exist for rotating trainees/fellows? This question allows us to collate information for trainees interested in Trauma. Thank you for your time.

## Appendix B See Table B1

**TABLE B1:** RACS Trauma Care Verification Program (TCVP) requirements for accreditation for each level of trauma facility and the minimum standards required to be classified within each category.

Level	Core capability	Role in trauma system	Typical hospital type
Level 1	Full spectrum trauma care: consultant—led 24/7; definitive care from resuscitation to rehab	Regional/national leadership; receives all major trauma; research, education, prevention, Quality Assurance	Large tertiary/quaternary centres
Level 2	Comprehensive clinical care equivalent to Level 1 but less academic/regional leadership	Supplements Level 1 in dense or regional populations; manages severe trauma	Large metropolitan or major regional hospitals
Level 3	Manages non major trauma; stabilises major trauma for transfer; limited definitive care	Feeder hospitals' stabilisation and selective definitive care	Regional/secondary hospitals
Level 4	Resuscitation and early stabilisation only; rapid transfer required	First point of care in rural/remote settings	Rural hospitals, community hospitals
